# Effect of Cyclical Bending and Rubbing on the Characteristic Impedance of Textile Signal Lines

**DOI:** 10.3390/ma14206013

**Published:** 2021-10-12

**Authors:** Jacek Leśnikowski

**Affiliations:** Faculty of Material Technologies and Textile Design, Institute of Architecture of Textiles, Lodz University of Technology, Żeromskiego 116, 90-543 Lodz, Poland; jacek.lesnikowski@p.lodz.pl

**Keywords:** textile signal lines, textile transmission lines, smart clothing, textronics, e-textiles, wearable electronics

## Abstract

This article presents the results of tests on the resistance of new textile signal lines to bending and abrasion. The textile signal lines are one of the most important parts of the electronic system incorporated into modern smart garments. The main application of the lines presented in this article is the transmission of digital signals or high-frequency analogue signals. The tested lines were made of fabrics with sewn paths made of electro-conductive fabric. The construction of a measuring stand for testing the electric properties of textile transmission lines is shown. This article presents the effects of bending and abrasion on the resistance of electro-conductive strips, which are one of the elements of textile signal lines. The article also presents the effects of bending and abrasion on the characteristic impedance of constructed textile signal lines. Statistical analysis of the obtained results is also presented.

## 1. Introduction

The textile signal lines are one of the more important parts of the electronic system implemented in “e-textiles”. The term “e-textiles” refers to common textiles with extended functionality. This extended functionality can be obtained, among other ways, by placing electronic circuits in the textiles. These textiles can perform various functions, e.g., monitoring human physiological parameters [1,2,3,4,5,6,7,8,9], ensuring communication, exchanging information [10,11], etc. For proper operation of such systems, each of its modules should be electrically connected to the other. These connections can be made with conventional or textile cables. The main disadvantage of using conventional cables is their high stiffness, increasing their perceptibility by the user of “e-textiles”. This significantly reduces the ergonomics of using such products. Textile lines do not have this disadvantage. Today, there are many methods for making textile signal lines that can replace conventional cables. These methods have been widely described in the literature [12,13,14,15,16,17,18]. Textile signal lines (TSLs) can be used to transmit not only constant or low-frequency signals but also high-frequency signals [17]. For example, they can be used as lines for the transmission of high-speed digital signals or lines to connect radio transmitters with textile antennas [19,20,21]. Requirements regarding the properties of lines intended for the transmission of high-speed signals are higher than in the case of other lines. In addition to resistance and current carrying capacity, the properties of this type of line are characterized by their characteristic impedance and s-parameters [22,23].

TSLs used in smart garments are subjected to various types of mechanical deformation during their use. During normal use in e-textiles, they may be subjected to stretching, bending, or abrasion. So far, no extensive research has been undertaken on the influence of various types of mechanical interaction on the transmission properties of TSLs. Nevertheless, these are very important issues because such lines should work properly not only in laboratory conditions but also in regularly used smart textiles. Lesnikowski and Kubiak [24] studied the changes in the characteristic impedance of selected types of TSLs during mechanical loading. These tests consisted of stretching the tested lines with a specified force and measuring the impedance profile after this stretching. As a result of the research, they [24] found that the tensile forces reduced the characteristic impedance of the line. They also found that the lines made of fabrics with a satin weave had the smallest impedance change under the influence of force loading. In turn, the lines with a substrate made of twill weave fabrics showed the largest impedance changes. TSLs placed in smart garments can not only be stretched; they can be bent and rubbed just as often. Therefore, this article presents the effects of cyclical bending and rubbing on the electrical properties of the selected type of TSLs.

## 2. Materials and Methods

For the tests, textile lines were made by sewing electro-conductive strips onto a non-conductive textile substrate. The method of making the line is distinguished by its simplicity and the materials used to build the line are widely available on the market; production of the line is possible based on the equipment in any sewing room.

**Figure 1:**
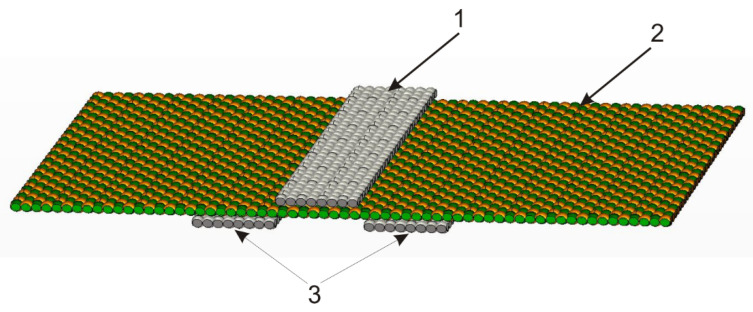
The construction of the tested lines.

**Figure 2:**
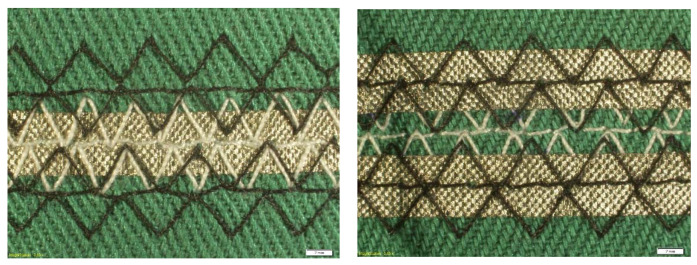
Examples of the view from the top (on the left) and the bottom (on the right) of the constructed line (white scale bar = 2 mm).

The construction of the tested lines is presented in Figure 1 and Figure 2.

The TSL consists of a signal path (1) (Figure 1) and two ground paths (3) made from electroconductive fabric. The signal path and ground paths are placed on opposite side of the substrate (2). The substrate of the line (2) was made from non-conducting fabric. Changing the distance between ground paths allows the characteristic impedance to be easily changed over a wide range. The signal and ground paths were stitched to the fabric substrate, as shown in Figure 2.

**Figure 3:**
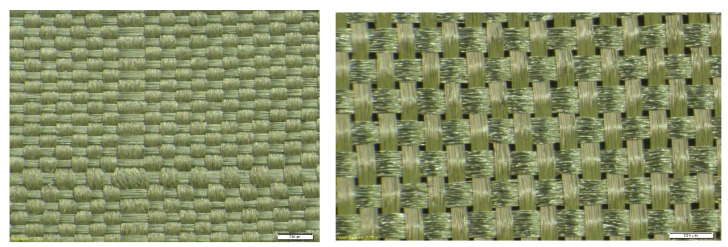
Details of Laird (on the left) and Ponge (on the right) electro-conductive fabric.

The basic parameters of the electroconductive fabrics used in this study are shown in Table 1. In Table 1, weft density determines the number of weft threads per unit length. The warp density determines the number of weft threads per unit length. The differences in texture or weaving details of both fabrics are shown in Figure 3.

The basic parameters of the fabrics used as a textile substrate of the tested lines are shown in Table 2.

The length of each line was equal to 25 cm, whereas its width was equal to 3 cm. The assumed width of the electroconductive tracks was 5 mm. For the tests, ten TSLs were made with substrates made of the fabrics described in Table 2. More information on the construction of the line can be found in [25].

The electro-conductive tracks of the TSLs used for further tests were made of Ponge fabric only. As a result of the tests presented later in the article, this fabric showed lower resistance to bending and abrasion. The use of Ponge to make electro-conductive paths in the tested lines was due to the desire to check whether a material with worse strength parameters would result in a line that bends and is abrasion-resistant to a degree that would allow its practical use.

### 2.1. Bending Stand

**Figure 4:**
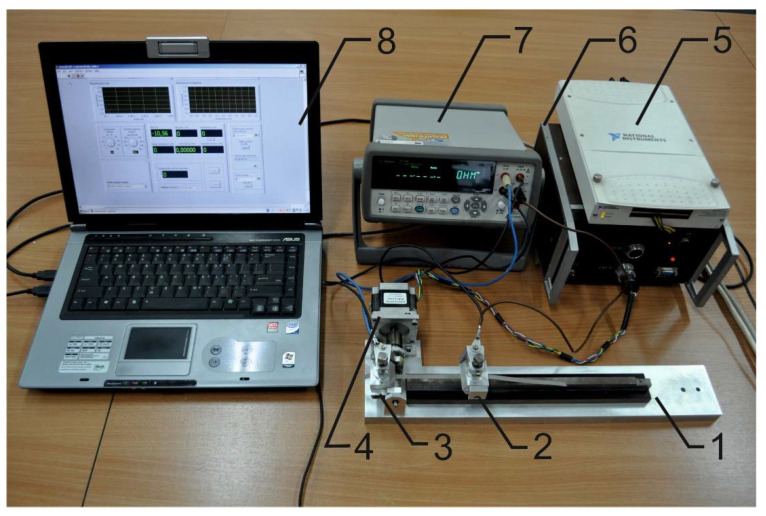
The stand for cyclical bending.

The stand for cyclical bending is presented in Figure 4. The stand consists of a holder for the TSL (1) equipped with a fixed (2) and a rotating clamp (3). The rotating clamp (3) is directly connected to the shaft of the stepper motor (4). The rotation of the shaft assures rotation of the rotating clamp (3) and cyclical bending of the tested line. The stepper motor is controlled by an electronic driver (6) and a data acquisition board (5) connected to the computer (8). The computer (8) is equipped with a virtual instrument program written using LabView software [26] and an Agilent 34410A multimeter (7). This multimeter can measure the resistance of the electro-conductive strips during bending.

**Figure 5:**
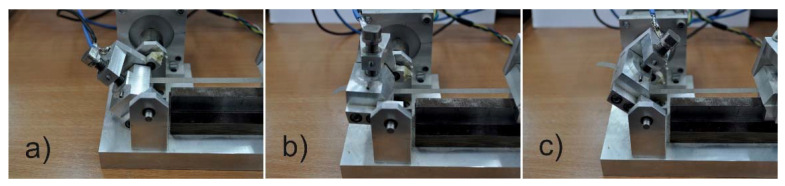
Stages of the bending process.

The virtual instrument allows changing of the speed and angle of bending. In the presented studies, the bending angle was assumed to be 90°. During one bending cycle, the tested line was bent 45° downwards (Figure 5a), then returned to its starting position (Figure 5b), then bent 45° upwards (Figure 5c), and then returned to its starting position (Figure 5b).

In the conducted tests, the centre of the line was assumed to be the bending place. The bending edge was perpendicular to the signal transmission direction.

### 2.2. Rubbing Fastness Stand

**Figure 6:**
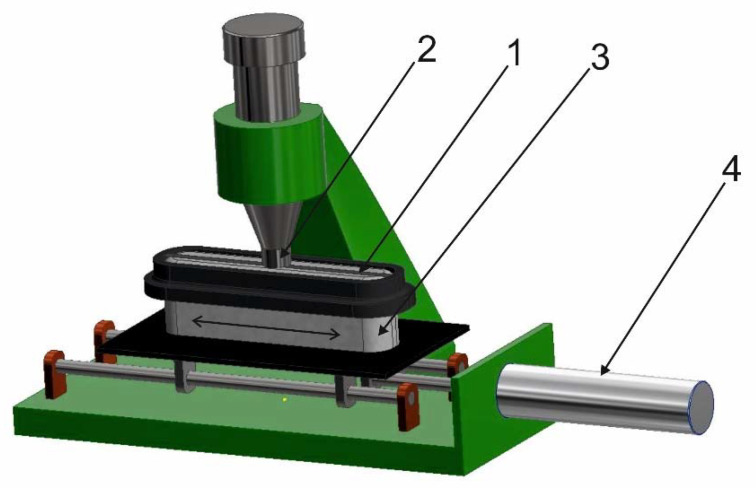
View of the experimental set-up for fastness testing of electro-conductive lines to rubbing.

The fastness of the electro-conductive properties of the tested strips and lines to rubbing was estimated using a motorised AATCC crock meter CBT507, a product of CBT (Poland), presented in Figure 6. The electro-conductive strip or TSL (1) (Figure 6) is placed on a movable table (3). The table movement is ensured by a computer-controlled pneumatic actuator (4). The tested sample (1) attached to the movable table (3) is rubbed with a rubbing finger (2). A white CO lawn (5 cm × 5 cm) is mounted on the rubbing finger (2). The diameter of the rubbing finger is 16.5 mm. Larger CO lawn dimensions (5 cm × 5 cm) result from the necessity to attach it to rubbing finger. The CO lawn is wrapped up around the rubbing finger. The pressure that the abrasive finger exerts on the sample to be rubbed is about 41 kPa.

### 2.3. Measured Parameters

All measurements presented in the article were carried out under standard testing climatic conditions at 20 °C and 65% relative humidity. A four-wire resistance measurement method and an Agilent 34410A meter (Agilent Technologies, Inc., Santa Clara, USA) were used to perform all resistance measurements. The accuracy of the resistance measurement with this meter is 0.0030 of reading + 0.0030 Ω.

#### 2.3.1. Bending

The first step of the study consisted of measuring the resistance vs. bending of the electro-conductive strips. These strips are one of the main elements of textile signal lines. Only electro-conductive strips made from Laird and Ponge electro-conductive fabric were tested. For bending, the test leads were connected to the clamps (2) and (3) (Figure 4) in order to measure the resistance of the tested device after each bending cycle. Based on these measurements, the relative resistance *R/R*_0_ was determined, where *R* denotes a resistance measured during the bending process and *R*_0_ is the initial resistance of the sample.

In the next stage, TSLs were tested and their characteristic impedance was measured. The characteristic impedance of the line is one of the main parameters characterizing its transmission properties in the time domain.

The required value of the characteristic impedance depends on the line application and the standard of the transmitted signals. For example, in radio transmission systems, cables with an impedance of 50 ohms are used to connect the transmitter with an antenna. The second important feature of the characteristic impedance is the invariance of its value along the entire length of the line. Lack of such invariance causes increased reflections of the transmitted signal and distortions of the signal passing through the line. To avoid such reflections, the impedance value should remain constant along the entire signal path containing the transmitter, transmission line, and receiver. The values of the characteristic impedance at each point of the line were obtained using the reflectometric method [27]. In this method, a generated voltage step is input to the tested line. The voltage step amplitude was 0.25 V. The voltage wave travels across the entire line. During the movement of the wave in the line, its partial reflection occurs on the impedance unevenness. The reflected wave returns to the beginning of the line, overlapping the generated voltage step. By measuring the voltage changes at the line input, the so-called line impedance profile can be determined. The impedance profile shows the characteristic impedance of each point of the line versus the distance of this point from the beginning of the line.

The stand used for measuring the characteristic impedance profile consisted of a Tektronix DSA 8200 sampling scope (Tektronix, Washington, DC, USA) equipped with sampling module 80E08. This module was connected to the tested TSL placed between two clamps, enabling the connection of its textile flat electro-conductive paths to the sampling module. More information regarding the stand and clamp construction can be found in [28,29].

The measuring process of each line included the following steps:

- fivefold measurement of the impedance profile of each of the tested lines;
- making 5000 bends of each of the tested lines;
- fivefold measurement of the impedance profile of each of the tested lines.

Steps 2 and 3 were repeated until 20,000 bends were reached. Thereafter, the bending process was continued for each line until 50,000 bends were reached.

**Figure 7:**
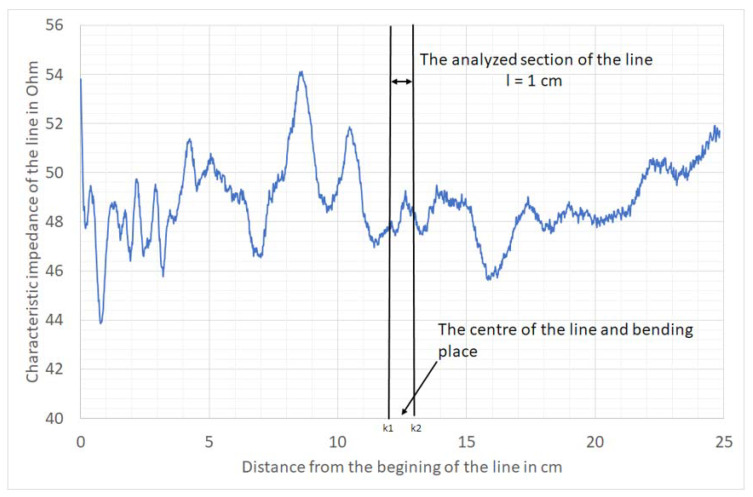
The average impedance profile of the F5 line with the Ponge substrate.

Between 20,000 and 50,000 bend cycles, fivefold impedance measurements were made every 10,000 bends. An example of the average impedance profile after the first step of the measuring procedure is presented in Figure 7.

Each obtained impedance profile is composed of n points. The bending place was the middle of the line. The middle section of the line, *l* = 1 cm long, was further analysed. To do this, from the *n* values of the characteristic impedance of the impedance profile, *k* values corresponding to the interval of length *l* (Figure 7) were selected. To perform statistical analysis, the average value and standard deviation of the characteristic impedance of this section were determined for each of the characteristic impedance profile measurements.

The value of the average characteristic impedance was calculated from the following equation:

(1) $$Z_{AV}=\overset{k2}{\underset{k=k1}{\sum }}\frac{Z_{k}}{k2-k1},$$

where *k*1 and *k*2 are the limits of the middle section of the line shown in Figure 7.

The value of the standard deviation of the characteristic impedance was calculated from the following equation:

(2) $$\sigma =\sqrt{\frac{\sum_{k=k1}^{k2}\left(Z_{k}-Z_{AV}\right)}{k2-k1-1}},$$

As a result, after each series of bends, five values of the characteristic impedance *Z_AV_* and five values of the standard deviation σ of the characteristic impedance were obtained. The standard deviation of the characteristic impedance is a measure of the quality of the line. As the standard deviation value increases, the transmission properties of the line deteriorate. This is due to the numerous reflections of the voltage wave on the impedance unevenness.

In the next step, the relative characteristic impedance *Z_x_/Z*_0_ was determined, where *Z_x_* denotes the average characteristic impedance measured after *x* bending cycles and *Z*_0_ is the average initial characteristic impedance before bending. Both of these values were calculated from Equation (1). Using Equation (2), similar calculations were performed to calculate the relative standard deviation of the characteristic impedance understood as *σ_x_*/*σ*_0_.

#### 2.3.2. Rubbing

In the abrasion resistance tests, tests of the electro-conductive strips and TSLs containing such strips were performed. For the electro-conductive strips, the rubbing procedure consisted of 2000 cycles of the finger along each electro-conductive strip. A white CO lawn was replaced every 200 cycles. The length of the abrasion was 10 cm. The width of the electro-conductive strips used in the abrasion test was the same as the width of the tracks used in the TSLs, i.e., 5 mm. The electrical resistance of each tested electro-conductive strip was measured in real time after each rubbing cycle. Based on these measurements, the relative resistance *R/R*_0_ was determined, where *R* denotes a resistance measured during the rubbing process and R_0_ is the initial resistance of the sample.

In the second part of the rubbing research, TSLs were tested. The same lines that were bent earlier were rubbed. The rubbing procedure consisted of 3000 cycles of the finger along the signal path (1) (Figure 1) of each tested TSL. After every 1000 cycles of rubbing, the tested TSL was removed from the AATCC crock meter and its characteristic impedance was measured. Based on the results of the characteristic impedance obtained after each abrasion stage, the values of the relative characteristic impedance and the standard deviation of the characteristic impedance were determined. The method and equations used were the same as for bending. When calculating the average impedance values in the case of abrasion, the entire line was taken into account, not only the middle part with a length of *l* = 1 cm as was the case with bending.

## 3. Results and Discussion

### 3.1. Bending

**Figure 8:**
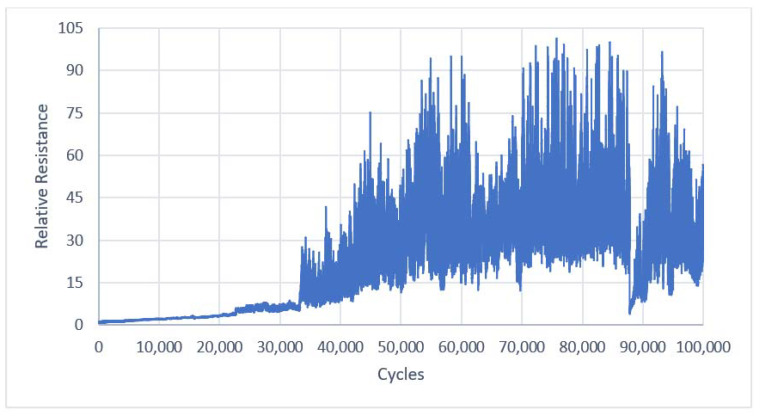
Average relative resistance of the electroconductive strips versus bending cycles for a Ponge/Soliani electro-conductive fabric.

**Figure 9:**
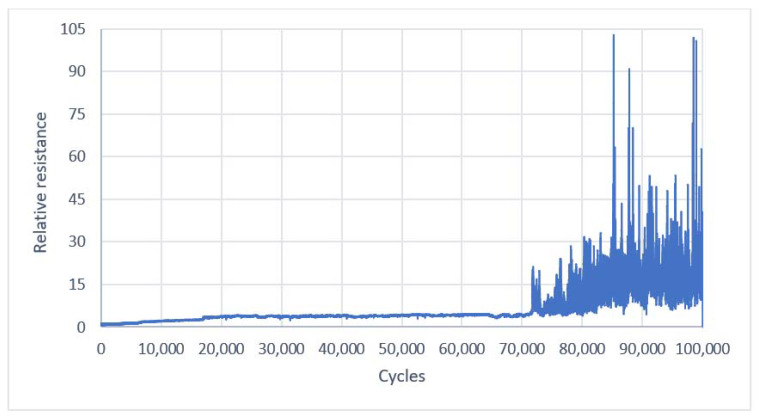
Average relative resistance of the electroconductive strips versus bending cycles for a 3050-525/Laird electro-conductive fabric.

During the tests, 100,000 bends of two Laird and two Ponge electro-conductive strips were made. The resulting two sets of resistance values for each of the tested fabrics were averaged. Next, the relative resistances were computed. Plots of the average relative resistance of the electro-conductive strips made from Ponge and Laird fabric versus bending cycles are shown in Figure 8 and Figure 9, respectively.

**Figure 10:**
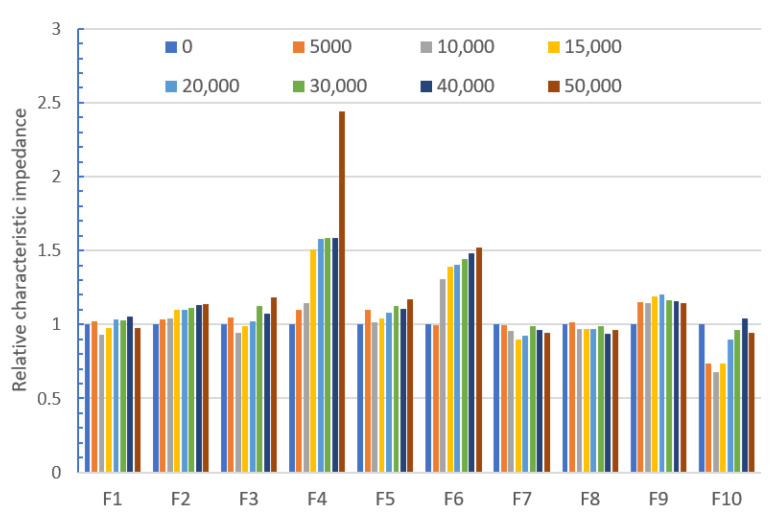
Relative characteristic impedance vs bending cycles for all tested TSLs with a Ponge substrate.

The results of the relative characteristic impedance for all bent TSLs are shown in Figure 10.

All the lines that were bent showed changes in the characteristic impedance during bending. In most cases, the relative impedance after 50,000 bending cycles does not exceed 1.2. This means that the tested lines are relatively resistant to bending. Only line F6 and line F4, in particular, showed an increase in the characteristic impedance above 50% of the initial value. For some applications, this may disqualify these lines. The conducted analysis showed that substrate parameters such as thickness, dielectric constant, and the other parameters presented in Table 2 do not influence relative characteristic impedance. Finding the reason for this will require further, more detailed research.

### 3.2. Rubbing

In the first step of the rubbing procedure, three strips made from Ponge fabric and three strips made from Laird fabric were rubbed.

**Figure 11:**
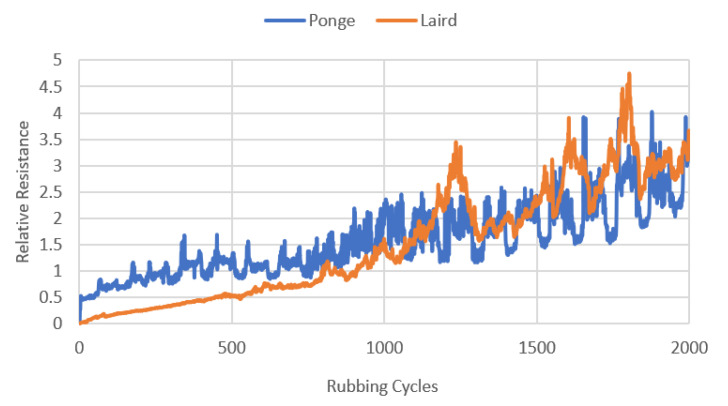
The average relative resistance of the electroconductive strips versus rubbing cycles for the Ponge and 3050–525/Laird electro-conductive fabrics.

A plot of the average relative resistance of the electroconductive strips versus rubbing cycles for the Ponge and Laird strips is presented in Figure 11.

**Figure 12:**
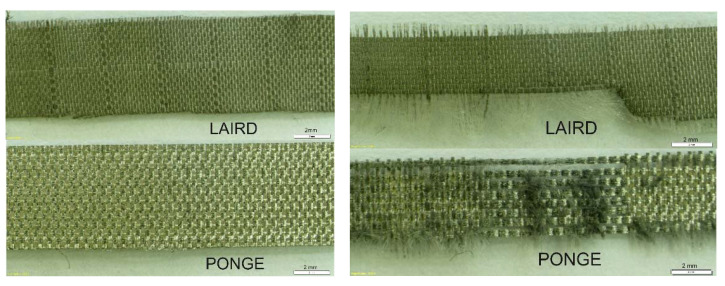
An example of the view of the Ponge and Laird strips before (on the left) and after (on the right) 2000 rubbing cycles (white scale bar = 2mm).

An example of the view of the Ponge and Laird strips before and after 2000 rubbing cycles is shown in Figure 12.

As can be seen from Figure 12, the damage to the strips is different. In the case of the Ponge strip, both the weft and warp threads are damaged. In the case of the Laird fabric, the damage consists of the loss of weft threads. The different nature of the damage may result from the different weaves of both fabrics. Despite the various types of damage, the changes in the resistance of both materials are comparable (Figure 11).

**Figure 13:**
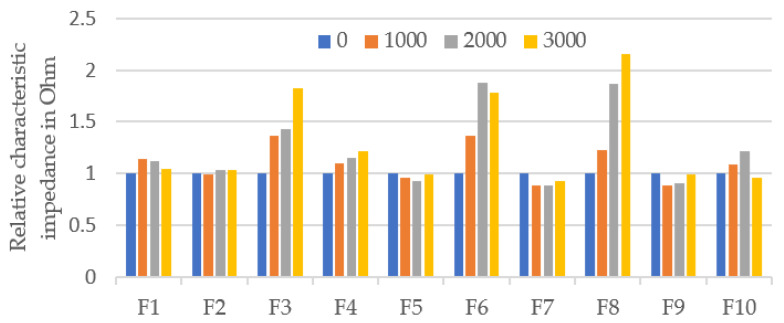
The average characteristic impedance of all TSLs with a PONGE substrate versus rubbing cycles.

In the next step of the rubbing procedure, TSLs were rubbed. The average relative characteristic impedance of all TSLs versus rubbing cycles is presented in Figure 13.

As can be seen in Figure 13, most TSLs show no significant impedance changes from abrasion. The exceptions are lines F3, F6, and F8, where the characteristic impedance increases significantly. The analysis of the properties of these lines showed that they were lines with a low thickness substrate.

## 4. Statistical Analysis of the Measurement Results

A statistical analysis of the obtained measurement results was also carried out. The goal of the statistical analysis was to determine whether bending/rubbing has a statistically significant effect on the value and variability of the characteristic impedance of the tested lines. Non-parametric tests were used instead of the multifactor analysis of variance (ANOVA). This was due to failure to meet the assumptions of analysis of variance, namely a lack of normality of distributions in groups determined by variables, and a lack of homogeneity of variance. The obtained measurement results are dependent variables, due to each successive group of measurements being made on the same successively bent or rubbed line. Therefore, for this purpose, the non-parametric statistical Anova Friedman test for dependent variables was used [30,31]. This non-parametric test is used to compare three or more matched groups. The effect of bending/rubbing on the relative characteristic impedance of the line and on the standard deviation of the characteristic impedance was checked. The test was repeated for each of the ten tested lines (F1–F10). For bending, the measurements resulted in eight groups of characteristic impedance measurements. These are groups containing five measurements made after 0, 5000, 10,000, 15,000, 2000, 3000, 40,000, and 50,000 bend cycles. For each group five relative impedance average values and five standard deviations were calculated based on the five raw impedance values. The method of calculating these values is presented in Section 2.3.1. For rubbing, the measurements resulted in four groups of characteristic impedance measurements. These are groups containing five measurements made after 0, 1000, 2000, and 3000 rubbing cycles.

The Friedman’s test is an ideal statistic to use for a repeated measures type of experiment to determine if a particular factor has an effect. For this test, the zero hypothesis *H0* assumed no effect of the tested parameter (bending or rubbing) on the characteristic impedance or standard deviation of the characteristic impedance. The null hypothesis is verified by a statistic built on the basis of the *Z* statistic:

(3) $$\chi^{2}=\frac{12}{Nk\left(k+1\right)}\overset{k}{\underset{i=1}{\sum }}R_{i}^{2}-3\left(k+1\right)N,$$

where *N* is the number of groups, *k* is the number of measurements in each group, and *R_i_* is the sum of the rank of the measurements for the *i*-th group. The value calculated from Equation (3) is compared with the critical value. If the obtained *χ*^2^ value is less than or equal to the critical value, then *H0* is retained. Otherwise, *H0* must be rejected. The Statistica software was used to perform the above test. In addition to the *χ*^2^ value, the Statistica software calculates a probability value shown as a *p*-value. The *p*-value is the value of the probability that the hypothesis *H0* is true. If the *p*-value is lower than the assumed significance level α, the assumed hypothesis *H0* should be rejected. In all tests, the significance level at α = 0.05 was assumed. The obtained test results for the characteristic impedance and standard deviation of the characteristic impedance are shown in Table 3 and Table 4, respectively.

Assuming a significance level at α = 0.05, based on the obtained *p*-values almost all of the tested *H0* hypotheses were rejected (*p* < α). This means that the bending and abrasion of the tested lines affected their characteristic impedance and its unevenness along the line. To more accurately determine after how many bending cycles the change in characteristic impedance was statistically significant, the Wilcoxon statistical test was used for two dependent variables. The Wilcoxon rank-sum test is commonly used for the comparison of two groups of non-parametric data. The Wilcoxon Signed Rank Test is the non-parametric equivalent of the *t*-test. The Wilcoxon procedure computes a test statistic W that is compared to an expected value. W is computed by summing the ranked differences of the deviation of each variable from a hypothesized median above the hypothesized value. With this test, the hypothesis that there were no differences between groups “0” and “n” was verified. The group “0” is the set of measurements before the bending/rubbing. The set “n” is the set of measurements after n bending/rubbing cycles. The Wilcoxon test was performed for each TSL and each “n” using the Statistica software. In this test, the Statistica software also calculates the probability (*p*-value) that the H0 hypothesis is true. Based on the obtained *p* values, the number of bending/rubbing cycles that caused a statistically significant change in the characteristic impedance was determined. The number of such cycles was also determined for the standard deviation of the characteristic impedance. The results are shown in Table 5.

In almost all cases, the statistical tests show that bending/rubbing had a statistically significant impact on the characteristic impedance of the TSL. The bending/rubbing also had a statistically significant impact on the standard deviation of the characteristic impedance of the line. This does not mean that lines of this type are not suitable for use in clothing. In practice, the final assessment of the resistance of lines to bending and abrasion depends on the type of signals transmitted, their frequency, or their spectrum width. In conditions of intensive use of clothing, these lines should not be placed in locations exposed to bending or friction.

## 5. Conclusions

In this article, the effect of cyclical bending and rubbing on the characteristic impedance of textile signal lines was presented. Two fabrics were used in the study: 3050-525/Laird and Ponge. The resistance tests to bending and abrasion showed that the 3050-525/Laird fabric is more resistant to bending than the Ponge fabric. The 3050-525/Laird fabric is also more abrasion resistant than the Ponge fabric for a small number of abrasion cycles (number of cycles <1000). Textile signal lines also were tested. The tests showed that the tested lines are more resistant to bending than to abrasion. The bending/rubbing had a statistically significant impact on the characteristic impedance of the TSL. The bending/rubbing also had a statistically significant impact on the standard deviation of the characteristic impedance of the line. This means that bending/rubbing affects the quality of the signal transmitted over the line. The conducted analysis also showed that lines with a thicker substrate are characterized by a higher abrasion resistance. The type of transmitted signal affects the applicability of the line in clothing. In the case of lines transmitting signals with a wide frequency spectrum, they should be placed in locations not exposed to bending and abrasion.

## Figures and Tables

**Table 1:** The basic parameters of the electroconductive fabrics used in this study.

Material	Trade Name/Producer	Thickness (mm)	Surface Resistivity (Ohm/sq)	Metal Amount (g/m^2^)	Total Weight (g/m^2^)	Weave	Warp Density (Yarns/cm)	Weft Density (Yarns/cm)
Nickel metallised polyester	Ponge/Soliani	0.15	Max. average 0.4	16	45–75	Plain	26	18
Ni/Cu Nylon Ripstop	3050-525/Laird	0.127	0.07	27–39	71–92	Twill	55	40

**Table 2:** Basic parameters of the fabrics used as a textile substrate of the tested lines.

Fabric No.	Raw Material	Weave	Dielectric Constant (1 GHz)	Tangent Loss (1 GHz)	Thickness (mm)	Surface Weight (g/m^2^)	Warp Density (threads/cm)	Weft Density (threads/cm)
F1	cotton	twill	1.825	0.049	0.62	287	30	19
F2	polyester	plain	1.351	0.005	0.36	158	56	28
F3	cotton	plain	1.688	0.040	0.29	114	29	23
F4	cotton	twill	1.847	0.044	0.31	201	36	12
F5	cotton	plain	1.631	0.032	0.38	89	25	21
F6	cotton	plain	1.551	0.011	0.30	85	26	14
F7	cotton	plain	1.857	0.045	0.40	102	24	32
F8	polyester	plain	1.361	0.007	0.25	150	36	22
F9	polyester	plain	1.924	0.006	0.53	275	24	21
F10	polyester	plain	1.477	0.005	0.34	145	64	33

**Table 3:** Results of Anova Friedman non-parametric tests for the characteristic impedance.

Fabric	Obtained *p*-Value for Bending	*H0* Hypothesis	Obtained *p*-Value for Rubbing	*H0* Hypothesis
F1	0.0012	Rejected	0.00285	Rejected
F2	0.0005	Rejected	0.00375	Rejected
F3	0.0002	Rejected	0.00285	Rejected
F4	0.0002	Rejected	0.00182	Rejected
F5	0.0003	Rejected	0.00698	Rejected
F6	0.0005	Rejected	0.00285	Rejected
F7	0.0009	Rejected	0.02627	Rejected
**F8**	**0.16702**	**Accepted**	0.00285	Rejected
F9	0.0034	Rejected	0.00285	Rejected
**F10**	0.0100	Rejected	**0.16045**	**Accepted**

**Table 4:** Results of Anova Friedman non-parametric tests for the standard deviation of the characteristic impedance.

Fabric	Obtained *p*-Value for Bending	*H0* Hypothesis	Obtained *p*-Value for Rubbing	*H0* Hypothesis
F1	0.00007	Rejected	0.00182	Rejected
F2	0.00085	Rejected	0.00182	Rejected
F3	0.00702	Rejected	0.00182	Rejected
F4	0.00002	Rejected	0.00182	Rejected
F5	0.00006	Rejected	0.00698	Rejected
F6	0.00046	Rejected	0.00357	Rejected
F7	0.00066	Rejected	0.00500	Rejected
F8	0.00046	Rejected	0.00182	Rejected
F9	0.00331	Rejected	0.00285	Rejected
**F10**	0.02974	Rejected	**0.06237**	**Accepted**

**Table 5:** The number of bending/rubbing cycles that caused a statistically significant change in the characteristic impedance/standard deviation of the characteristic impedance.

Fabric	Characteristic Impedance (Bending Cycles)	Characteristic Impedance (Rubbing Cycles)	The Standard Deviation of the Characteristic Impedance (Bending Cycles)	The Standard Deviation of the Characteristic Impedance (Rubbing Cycles)
				Number of rubbing cycles
F1	5000	1000	20,000	1000
F2	5000	2000	5000	1000
F3	5000	1000	20,000	1000
F4	5000	1000	10,000	1000
F5	5000	1000	5000	2000
F6	10,000	1000	40,000	1000
F7	10,000	1000	5000	1000
F8	50,000	2000	5000	1000
F9	5000	1000	30,000	1000
F10	10,000	2000	15,000	3000

## Data Availability

Experimental methods and results are available from the authors.
